# Patient experience of the process to diagnosis of chronic limb‐threatening ischaemia: A qualitative study

**DOI:** 10.1002/jfa2.12042

**Published:** 2024-07-17

**Authors:** Eleanor Atkins, Ian Kellar, Panagiota Birmpili, Jonathan R. Boyle, Arun D. Pherwani, Ian Chetter, David A. Cromwell

**Affiliations:** ^1^ Clinical Effectiveness Unit Royal College of Surgeons of England London UK; ^2^ Hull York Medical School Hull UK; ^3^ University of Sheffield Sheffield UK; ^4^ Department of Vascular Surgery Cambridge University Hospitals NHS Trust and Department of Surgery University of Cambridge Cambridge UK; ^5^ Staffordshire & South Cheshire Vascular Network Royal Stoke University Hospital Stoke‐on‐Trent UK; ^6^ Department of Health Services Research and Policy London School of Hygiene and Tropical Medicine London UK

**Keywords:** chronic limb threatening ischaemia, patients, vascular surgery

## Abstract

**Introduction:**

Delays exist at each stage of the chronic limb‐threatening ischaemia (CLTI) care pathway, but there is little known about patient factors influencing delay to diagnosis of CLTI. This study explores the experiences and perceptions of patients recently diagnosed with CLTI.

**Methods:**

A qualitative interview study was conducted. Sixteen participants underwent semi‐structured interviews. Reflexive thematic analysis was performed on the data, aiming to understand factors which can influence delay in the CLTI care pathway.

**Results:**

Five interrelated themes were developed: CLTI is a devastating condition; Reluctance to ask for help; When we are empowered we get better care; Luck plays a role in the process to diagnosis; and Vascular units can do better, comprising sub‐themes of information transfer—consider communication and arterial versus non‐arterial centres—proximity isn't everything.

**Conclusions:**

The five themes generated from the interview data describe factors relevant to delay given meaning by participants who have lived experience of CLTI. Theme content should be noted by clinicians, commissioners and providers looking to improve care pathways for patients with CLTI. The importance of awareness for the public, patients and clinicians linked ideas in some themes and interventions to raise awareness should be considered.

**Abbreviations**
CLTI: chronic limb‐threatening ischaemia
COREQ: consolidated criteria for reporting qualitative research
ED: emergency department
GP: general practitioner
NHS: National Health Service
PAD: peripheral arterial disease
PIS: participant information sheet

## 1 INTRODUCTION

Chronic limb‐threatening ischaemia (CLTI) is the end stage of peripheral arterial disease (PAD). It is estimated that CLTI is diagnosed in 500 to 1000 patients per million in the United Kingdom (UK) each year [1]. Symptoms include non‐healing ulceration, gangrene and/or severe pain in the affected limb [2]. A diagnosis of CLTI puts the patient at risk of amputation and death, with over 40% of patients with tissue loss losing a limb or dying at 2 years [3]. One treatment for CLTI is revascularisation, carried out by vascular surgeons. This significantly reduces the risk of losing a limb or dying, with the mortality benefit sustained to at least 4 years [3].

There are delays at each stage of the CLTI pathway and missed opportunities for diagnosis of CLTI in primary care [4, 5]. Delays can have severe consequences, including a higher risk of limb loss and death [6]. Little is known about the patient experience of CLTI, with published studies focussing on complications of diabetic foot disease [7, 8, 9, 10, 11]. This is a cohort of patients with some overlap but important differences from patients with CLTI. Where literature exists on patients with CLTI, it explores their concerns and values around treatment, or perception of their own body and does not expand on care pathways [12, 13].

Vascular care in the UK is provided in a network model. Each network comprises an arterial centre, where the vascular unit is based with 24/7 vascular cover, and associated non‐arterial centres which can provide outpatient clinics and diagnostic imaging but not inpatient vascular care. Shared pathways of care exist between arterial centres and non‐arterial centres, aiming to provide equal access to vascular services [14]. Current pathways in place for patients with suspected CLTI have previously been demonstrated in a process mapping study [15].

Patient understanding of PAD and consequent delay to first presentation has been implicated in previous interviews with clinicians as a cause for delay to treatment [16, 17]. Despite this, there is little investigation in the literature into how and why the patient's perspective may affect diagnosis and assessment of CLTI. Delays in management of CLTI lead to significant consequences for the patient and their family, such as mortality and limb loss. Whilst national guidance provides recommendations to standardise time‐to‐revascularisation [18], it focuses on care pathways under the control of vascular units, rather than the time prior to referral. This is an important part of the patient pathway which should be considered in efforts to improve outcomes in the future. The aim of this study was to explore the experiences and perceptions of patients diagnosed with CLTI, focussing on their process to diagnosis, in order to understand factors potentially associated with delays in the process, as told by patients themselves.

## 2 METHODS

A qualitative interview study was performed. Qualitative research enables understanding of how the world is viewed by research subjects [19]. The authors sought to explore individual participants' experiences and perceptions of achieving their diagnosis of CLTI and define common meaning across the cohort. A critical realist framework underpinned the analysis, appreciating that multiple experiences and perceptions of a single reality exist. Coding and theme development was based on meaning, as opposed to frequency, in keeping with a ‘big Q’ qualitative paradigm [20]. Reflexive thematic analysis, a qualitative method developed by Braun and Clarke, was used to explore and interpret the dataset, allowing the authors to develop and tell a story of its patterns of meaning [21]. The consolidated criteria for reporting qualitative research (COREQ) guided the reporting of this study (Additional file 1) [22].

### 2.1 Identification and recruitment of participants

Participants were recruited by vascular surgery clinicians at seven NHS Trusts in England. These Trusts were previously involved in a process mapping project, meaning the authors were aware of the context in which the participants were referred, assessed and managed [15]. Eligible participants had been diagnosed with CLTI in the past year, had no cognitive impairment and were able to speak English. The purposive sampling strategy aimed for maximum variation in age, gender, diabetes status and patient location in the vascular network (near an arterial or non‐arterial centre). The diabetes status and patient location within the network have previously been identified as areas of inequality in care pathways for suspected CLTI [15].

Potential participants were consented by a vascular clinician involved in their care for the sharing of their contact details with the research team. Participants were then contacted by the research team, and if interested, given more information on the study in the form of a participant information sheet and the opportunity to ask questions. Written consent was obtained from each participant prior to interview and consent confirmed verbally before and after. Ethical approval for this study was gained from the NHS Health Research Authority and the South Yorkshire Research Ethics Committee (22/YH/0290).

### 2.2 Interviews

Semi‐structured interviews were carried out by EA, a female vascular surgeon with experience in qualitative interviewing. All interviews were carried out over the telephone. A topic guide consisting of open questions was used as a framework for the interviews, with prompts used where necessary (Additional file 2). It was iteratively altered as interviews progressed.

A reflexive diary was kept throughout the process by EA, with individual reflections recorded after each interview. This enables reflexive thematic analysis, where the researcher's subjectivity is acknowledged and informs the analysis [21].

### 2.3 Analysis

All telephone interviews were audio recorded, transcribed verbatim, anonymised and imported into a qualitative software package (NVivo) to aid data analysis. As per Braun and Clarke's six phases of thematic analysis, immersion in the data took place both with the audio recordings and the transcripts, with initial noting of observations, questions and interpretations. This allowed EA to become extremely familiar with the data. Formal inductive coding was carried out by EA, followed by two cycles of re‐coding. Here, the participant transcripts were worked through line‐by‐line, with utterances relevant to the research question being allocated labels, or codes, to capture single meanings. Both semantic and latent codes were identified, referring to explicit or implied content in the data. Re‐coding involved going back through transcripts already coded and developing the previous codes according to the coding in the rest of the dataset. Candidate themes, named clusters of codes which shared a core idea, were generated initially, then developed and revised following discussion with the author team and re‐engagement with the original data. This ensured themes were adequately telling the story of a pattern of meaning across the dataset. Themes were then refined, defined and named. Links between the themes were considered and discussed with the wider author team.

## 3 RESULTS

Sixteen individuals who had been diagnosed with CLTI in the year preceding participated in telephone interviews. Participants were aged 59–80 (mean 67.4) years. Two were female. Four participants had diabetes (Table 1). None dropped out or rescinded consent at a later stage.

**TABLE 1:** Participant characteristics.

Patient code	Male/female	Age	Diabetes	Arterial/non‐arterial centre
A	M	59	No	Arterial
B	F	63	Yes	Non‐arterial
C	M	63	No	Arterial
D	M	66	Yes	Arterial
E	M	74	No	Arterial
F	M	69	Yes	Non‐arterial
G	M	67	No	Arterial
H	M	65	Yes	Arterial
I	M	60	No	Non‐arterial
J	F	72	No	Non‐arterial
K	M	74	No	Arterial
L	M	80	No	Non‐arterial
M	M	59	No	Arterial
N	M	72	No	Non‐arterial
O	M	65	Yes	Non‐arterial
P	M	71	No	Non‐arterial

Interviews lasted from 38 to 61 (mean 47) minutes. Reflection on the content of our dataset found the interview data adequately rich to fulfil our research aim, according to the concept of information power [23].

**FIGURE 1:**
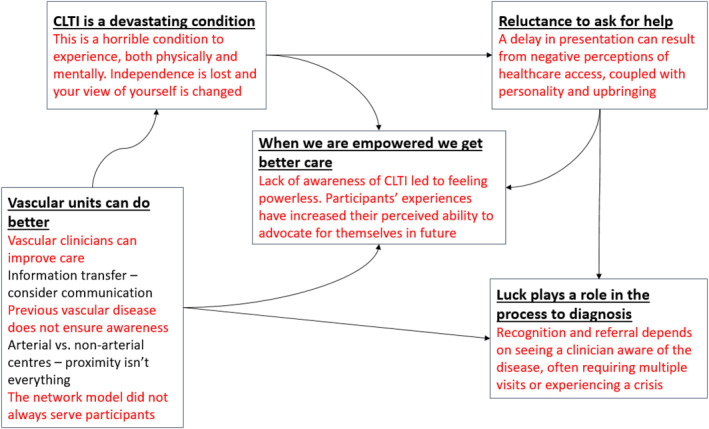
Diagram of themes.

Reflexive thematic analysis of the interview data led to the development of five key interrelated themes: CLTI is a devastating condition; reluctance to ask for help; when we are empowered we get better care; luck plays a role in the process to diagnosis and vascular units can do better (Figure 1).

### 3.1 Theme 1: CLTI is a devastating condition

The theme ‘CLTI is a devastating condition’ reflects the participants' descriptions of CLTI as a condition with multiple effects on every aspect of their lives. Physical symptoms were severe and disabling, affecting mobility and sleep.

They cramped, and they locked. It’s like your legs have been locked, and when you get up, you’re sort of hopping around, trying for the pain to go away, like. And that was it virtually every day, every night, like, you know.

Participant K, 74M

Whenever I tried to walk anywhere, the pain was like pretty intense, and resting, well, it wouldn't let me rest. It would be painful.

Participant C, 63M

I was having to sleep in a chair, a dining chair, with my feet down, which was uncomfortable!

Participant P, 71M

I was having an hour and a half, two hours sleep a night.

Participant G, 67M

To me, it's a lifetime when you're waiting a week for an appointment, when your toe’s black, and it's smelly. A week’s a lifetime.

Participant H, 65M

The severity of symptoms led to mental health challenges, including thoughts of self‐harm and suicide. They changed how participants viewed themselves.

So I just came home. Basically went on to smoke myself to death. Well, I didn’t think there was nothing to live for.

Participant G, 67M

See, I'll go up on a railway line one night and put my leg on there and, you know, hope for a train to go over it.

Participant K, 74M

I’ve come to realise that I'm not a young man anymore. I'm more susceptible to disease and things going wrong. It has changed my outlook as well.

Participant D, 66M

Symptoms led to a loss of independence. Participants described being unable to carry out normal activities of daily living, including socialising and relying on friends or family for help. Participants found this difficult, having previously been independent. Predominantly female family members were involved in helping their relative struggling with CLTI symptoms.

And it's hard sort of accepting, nope, you’ve now fallen into the net of being in a bit of need.

Participant O, 65M

It meant that, you know, I wasn't going out very much at all, and even to do shopping, I was getting shopping delivered, etcetera, etcetera. And I was getting picked up by a member of the [workplace] staff and brought home again.

Participant P, 71M

I didn't eat properly because I couldn't stand and cook anything, you know?

Participant B, 63F

Well, my daughter was cooking me dinners, and I was like… By the time I came into hospital, she was cooking me dinners. She was doing the hoovering, doing everything.

Participant G, 67M

CLTI also impacted on working life, leaving participants with difficulty carrying out their normal duties in their employment, which could lead to financial consequences.

I was trying to go to work, and if it wasn't for my workmates I wouldn't really have made through it, because they covered for me. While I just had a good rest, sat at a desk, did computer stuff, paperwork.

Participant D, 66M

I'm now still off work. You know, we’re selling stuff, you know, to basically keep the wolf from the door.

Participant I, 60M

Another struggle for participants was the lack of an initial diagnosis. They didn't know what was wrong with them and this was difficult to manage mentally, leading to frustration.

More than anything, I think, yeah, not knowing what it was, you know, infuriated me a bit.

Participant A, 59M

Well, once we knew what was happening, what was wrong. That was the big thing. It was the not knowing. And it wasn't getting any better.

Participant I, 60M

### 3.2 Theme 2: Reluctance to ask for help

The reluctance of participants to ask for help was described in terms of a personality type or upbringing, coupled with a negative perception of the process of accessing healthcare or not wishing to waste anyone's time. Participants described themselves as stoic generally.

But I, to be honest, I don't like hospitals because I'm one of them, I’m sturdy.

Participant N, 72M

You know, I'm a fairly strong character, I think.

Participant M, 71M

How participants were brought up, and consequently how they saw their role in society, affected their willingness to ask for help.

We were always contributors, we were never takers, and, you know, we had pride in that. I don't know what work ethic it was Mum and Dad had, that they put into me, but it was a case of, no, we're supporters, we're not vulnerable, we're not needy. You know, we look after ourselves, we'll deal with it.

Participant O, 65M

The NHS was seen to be under pressure, with healthcare providers perceived to be having plenty to deal with. Participants didn't want to add to that pressure unnecessarily.

The NHS [National Health Service] are really busy, so I just left it and left it.

Participant A, 59M

And then over the years, with the way that they, like, the way that they’ve been put under the cosh, you don't want to torment them anymore than what you have to.

Participant G, 67M

You know, I don't want to waste their time. Their time, it is as precious as anybody else's.

Participant C, 63M

Participants anticipated difficulty, discomfort or futility in accessing care, based on previous experiences accessing care in the community, which put them off seeking help.

Well, the GP [general practitioner] surgery’s just ‐ it's a nightmare. So, unfortunately, you know, unless I'm absolutely desperate I tend not to use them.

Participant M, 59M

…but I certainly would be able to speak to my GP ‐ if I can get an appointment with my GP, of course, because that in itself is like gold dust, trying to get a hold of an appointment.

Participant B, 63F

Wasting time all the time. Sitting around, waiting. Trying to get… You can’t take a nap. You can't close your eyes for a minute, because somebody's gonna pop into you. And I was always watching my foot, you know, where people are pushing trollies past you. It's hectic. It’s mayhem.

Participant E, 74M, referring to the emergency department (ED)

I haven't gone back, because if he [vascular surgeon] said there was nothing they could do, I thought, well, what can the GP do?

Participant K, 74M

Other things were also going on in their lives, which participants placed more importance on than their own health.

Because I couldn't walk far, because I had pain. But I put it all to one side, didn't do anything about it because I was struggling with my husband at that time.

Participant J, 72F

### 3.3 Theme 3: When we are empowered we get better care

This theme describes the ability of participants to demand better care if they are aware of the likely cause of their symptoms and are able to articulate this to their clinicians.

Look. If I hadn’t taken action myself, and spoken to the vascular nurses, who I’d been under at [arterial centre], who knew me, I think I'd have still been stuck at home. They knew all my history, had all my notes, and that sort of prompted things into action, for me.

Participant J 72F

Participants described a lack of personal and public awareness of PAD including its severity, urgency and consequences, leading to a feeling of powerlessness during their process to diagnosis.

And it was after that that I noticed that I couldn't walk very far. And I just put that down to the lack of exercise and age. I was getting ‐ my legs were starting to really ache badly after, you know, after a couple of hundred metres.

Participant M, 59M

I just didn’t have a clue whatsoever what was going on, really […] I didn't realise it was that serious.

Participant F, 69M

But what could I say to them? You go to the doctors', and they tell you what they think. I can only say, it's hurting, and they say, don't be a wuss. You know what I mean?

Participant L, 80M

The importance of advocacy was expressed by participants, whether that is advocating for oneself, or having friends, family or trusted clinicians that can fulfil that role.

No, you know, and I virtually insisted that I wanted to go to hospital, like, and have it checked out like, you know.

Participant K, 74M

Because I say, I feel, well, I'm quite articulate, and can fight my corner, but there's a lot that can’t. And that’s a bit of a worry, you know. They just accept, or wait so long, and you know…

Participant J, 72F

The experience of CLTI that participants have lived through has enabled them to feel more competent to advocate for themselves in the future.

Well, I’d recognize the signs sooner, so that would be a bonus. I mean, I would probably demand an earlier referral.

Participant B, 63F

Participants reported unwillingness to use the internet to search for information, preferring face‐to‐face advice from experts. This was variously due to a lack of access to the internet, a lack of trust in the information found online and a perception that information found may increase the perceived severity of their symptom.

I don't go on computers to investigate this, that and the other. I’d rather a face to face. And be told point blank. And not, it's possible… It could be… Whereas… And you’re reading between the lines and you think you've got everything! So no, I don't go on to websites and things like that.

Participant E, 74M

…because if you're gonna worry, you can make yourself worry a hell of a lot more if you go onto Google, can’t you?

Participant H, 65M

### 3.4 Theme 4: Luck plays a role in the process to diagnosis

Luck was required for the participant to come across a clinician able to recognise and refer to CLTI appropriately, or for an event to occur to precipitate diagnosis.

And then the chance observation at my local GP, with their review nurse, who says, go on, let’s have a look at your foot. And what's this on your toe? Right, you’d better go and see them…

Participant O, 65M

Little awareness of CLTI symptoms and referral pathways was demonstrated by primary care and emergency department (ED) clinicians in the experiences of our participants. Participants frequently required multiple visits to clinicians, with minimal continuity of care and it was often by chance that a clinician was seen who was able to recognise and refer appropriately. Diagnosis of gout or arthritis were often made and if a wound was present there was often a period of dressing it without investigating underlying causes of poor healing.

So back to the doctor. She decided to take blood tests to establish what the problem was. There was a suspicion of gout, or arthritis.

Participant C, 63M

So when I went to hospital, [non‐arterial centre], I got this Asian doctor and he said to me, it’s gout. Take ibuprofen.

Participant N, 72M

Right, [nurse 2] dressed it, [nurse 1] dressed it. [Nurse 1] mostly dressed it. She wasn't happy about it, but she wasn't sure what to do. She spoke to plastic surgery people, and this, that and the other. Then there was two or three at [town 3], when they couldn’t get me in at [town 2] or [town 1].

Participant I, 60M

Because I saw three GPs, all different ones. We lost our original one. I think he would have been better at it. You know, because he… If you look at a person three times, you see a difference. But if you see three different people, it's a—you don't get the same effect. So it was just unlucky I got three different GPs.

Participant L, 80M

And then after about two weeks, two or three weeks, one of the nurses said, I'll do a Doppler, and did a Doppler on my leg. And that's when she referred me. And that's where she found out that my leg was, you know, 50% below sufficient.

Participant P, 71M

And it got to March, I collapsed. I went to [non‐arterial centre], they took me to [non‐arterial centre], and the doctor who saw me that time spotted it straight away.

Participant N, 72M

A diagnosis could be precipitated by a crisis, seen as lucky by the participant.

But I was lucky, in a way, to have that ulcer on my heel, otherwise I’d have just carried on as normal.

Participant P, 71M

### 3.5 Theme 5: Vascular units can do better

This theme comprises two sub‐themes, focussing on specific points for improvement. It communicates where vascular units can improve the care they provide to avoid delays. Vascular pathways were often described as slower than recommended time‐to‐revascularisation targets.

The communication possibly… it seems to take, yeah, we want you to see the surgeon. Well, they can see you in three weeks. Well, you know, when you've got the black toe, I think you need to be seen quicker sometimes.

Participant H, 65M

#### 3.5.1 Sub‐theme 1: Information transfer

Some participants reported previous contact with vascular surgery, with long‐standing histories of PAD or previous experiences of CLTI. A lack of awareness was evident, however, even in these cases, indicating vascular surgery communication with the patient had not been effective.

EA: And back when they said, ohh there's nothing else we can do. Did they give you any symptoms to watch out for, or any sort of conditions where they might do something?

Participant F, 69M: No. None whatsoever.

Despite recent experiences, participants lacked understanding about the pathology of PAD and treatment decisions made by vascular clinicians.

Yes, I became aware that I had issues. Which I always found peculiar because at the time I was going in for that, and they were all there wanting to cannulate in my arms, and obviously for the intravenous and all the other bits and pieces, every nurse that I saw said, what fabulous veins you’ve got!

Participant O, 65M

So I almost have that feeling, that it would have been better… Prevention rather than cure would have been a good thing. If they’d acted two years ago, instead of leaving it until it actually gets infected this year.

Participant H, 65M

Referral pathways did not seem to have been made clear by vascular networks to primary care clinicians, with a lack of awareness of local processes described.

And to cut that story short, the GP said, all I can do for you is ring 999. So that's what happened.

Participant J, 72F

And then I went back a week later, and saw him again. And I said, what's happening about this hospital appointment? Because I really can't carry on like this for much longer. And he said ‐ and by this time the toes were really quite purple at this point. And he said that they'd written back to him and they wanted more information, and they weren't sending out an appointment yet.

Participant B, 63F

#### 3.5.2 Sub‐theme 2: Arterial versus non‐arterial centre—proximity isn't everything

Differences between arterial and non‐arterial centres were described by the participants with experience of both. The arterial centre was often seen as a better hospital than non‐arterial centres.

Basically, just, because I know, I think it's a better hospital than [non‐arterial centre].

Participant I, 60M

The network model was highlighted as a cause for delay, with issues transferring participants for review and few opportunities to see urgent referrals at the non‐arterial centre.

It was just hard for them to get transport. That was where I got stuck at [non‐arterial centre] for longer when [arterial centre] were saying to [non‐arterial centre], well, we’ve got the bed waiting. Where is she?

Participant J, 72F

They looked to see if there was anything before that day, but apparently that clinic was only held one day a week, on Wednesday at [non‐arterial centre], and there was nothing.

Participant B, 63F

Participants were willing to travel to the arterial centre and it was described as easier to get to than some network non‐arterial centres. Accommodations made to increase accessibility helped this.

It's probably slightly easier bus wise, although it's a longer journey, to get to [arterial centre] than it is to try and get to [non‐arterial centre] from here, because I don't think there’s a direct bus anymore.

Participant B, 63F

No, because even the night before the operation, I was given free accommodation at the hospital. So there was no problem at all, because they wanted me there at 7 o'clock in the morning. And to get from [non‐arterial centre] to [arterial centre] at that early hour, I don't think I would have made it.

Participant F, 69M

## 4 DISCUSSION

This qualitative study has explored the experiences of patients recently diagnosed with CLTI. The participants in this study have allowed us to increase our understanding of the care pathway for CLTI from a patient perspective and recognise those factors relevant to delays in the process directly from lived experience of CLTI diagnosis. In agreement with previous suggestions in the literature [8, 16, 17], participants described a lack of awareness of PAD and CLTI, its urgency, severity and potential consequences. In addition, the themes of ‘the role of luck in the process to diagnosis’ and ‘when we are empowered we get better care’ describe a lack of awareness of clinicians in primary care and EDs when participants presented, requiring either luck or patient knowledge and advocacy to ensure a diagnosis of suspected CLTI is considered. A lack of awareness of CLTI across healthcare professionals has previously been identified as a barrier to timely referrals in qualitative interviews with vascular surgery clinicians [8, 17], and missed opportunities to identify and refer CLTI have been described in a primary care database study [4], supporting our participants' perceptions. There is little evidence to suggest the most effective way to educate other clinicians, especially when they are dealing with high pressures throughout the healthcare system. There is an opportunity for improvement here, not only just to improve care for patients with suspected CLTI, but to add to the educational literature.

Some participants were reluctant to ask for help, as described in Theme 2, which is reflective of older adults' desire to meet their needs without assistance [24]. Access to healthcare is complex, depending on interplay between individuals and healthcare services [25]. Socio‐economically disadvantaged people are both more likely to be diagnosed with CLTI [26] and manage their health in a series of crises [25]. However, previously reported barriers preventing attendance for health promotion or prevention such as financial costs [27] or lack of other resources such as transport [28] were not described by our participants. They indicated instead that in being reticent to attend healthcare services they were reducing pressure on the NHS, which aligns with more recent findings [29]. Improving this is a challenge whilst there remains high pressure on NHS services, but suggestions have included improving information provision and building better connections across the health and care system [29].

Some of the barriers reported by participants are in the control of vascular surgeons, as described in the theme ‘vascular units can do better’. This links with the previously discussed lack of awareness, as participants demonstrated that even following diagnosis of CLTI, their understanding of the condition was poor. This is a common problem, with patients leaving hospital often unaware of their diagnosis and treatment plan [30, 31]. Our data indicates participants were unlikely to use the internet to research their symptoms and in addition, the internet cannot be relied upon to provide good quality information [32, 33]. CLTI is associated with poor health literacy [34]. This, in combination with our findings and the fact that CLTI has a significant risk of recurrence [35, 36], suggests vascular clinicians need to optimise the delivery of information to patients with CLTI. Education of patients can lead to fewer recurrences in diabetic foot ulcers, a similar condition [37]. A change in vocabulary has previously been suggested in CLTI care, with the use of the word ‘remission’ to signpost high rates of recurrence, aligning with language used in cancer management [38].

There are well recognised pressures on the NHS, as described by participants, which are unlikely to improve in the current context of increasing patient complexity and funding shortages [39, 40]. Ensuring primary care and ED clinicians are aware of local referral and escalation pathways for CLTI will reduce multiple visits to primary care and EDs, relieving pressure and reducing delay to treatment, which is associated with worse outcomes for mortality and limb loss [6]. Co‐production of care pathways with primary care clinicians, especially community nurses and podiatrists who are involved in lower limb wound care, would ensure that these groups are integrated into the care of patients with CLTI following diagnosis to reduce the burden on vascular surgery services. Empowering patients with known PAD to recognise signs and symptoms and refer themselves where such services are available could have the same effect [41].

### 4.1 Strengths and limitations

This qualitative interview study has explored the experience of participants diagnosed with CLTI in the preceding year. This is an appropriate study design, given little existing evidence in the literature. Participants were recruited nationally and selection prioritised variation in terms of comorbidity and location within the vascular network, ensuring different experiences were included. Notwithstanding this, our participants will not reflect all experiences of CLTI, especially as our cohort was relatively young and some patients with CLTI would be excluded from participation due to comorbidity and inability to speak English. Our findings are also unlikely to be transferrable to fee‐for‐service healthcare models, or beyond the UK. EA, the researcher who carried out interviews and analysis, is a vascular surgeon and used her subjectivity in this area to develop themes presented. This unique analysis is a hallmark of reflexive thematic analysis and should be embraced. An alternative researcher, however, with different assumptions and background, may have generated differently situated knowledge.

## 5 CONCLUSIONS

The themes generated in this study suggest that whilst CLTI is a distressing condition for patients, they are reluctant to ask for help due to perceived pressure on the NHS and their personality or upbringing. Once they do present, their symptoms are often not recognised as CLTI, and they undergo convoluted routes into vascular surgery assessment, often dependent on chance. Participants felt that had they been empowered by increased awareness of the condition and/or advocated for, they would have accessed care sooner. The final theme recognises the contribution of vascular surgery systems and processes to delay in care pathways. In addition to working with other stakeholders such as referring clinicians and those receiving referrals and assessing patients with suspected CLTI, these results should be considered in the design of interventions to improve care pathways for CLTI. The thread of awareness running through these themes has been highlighted as particularly important in the experiences of participants who have lived experience of CLTI.

## AUTHOR CONTRIBUTIONS

**Eleanor Atkins**: Conceptualization, data curation, formal analysis, methodology, writing – original draft, writing – review and editing. **Ian Kellar**: Conceptualization, formal analysis, methodology, supervision, validation, writing – review and editing. **Panagiota Birmpili**: Conceptualization, data curation, formal analysis, writing – review and editing. **Jonathan R. Boyle**: Conceptualization, supervision, writing – review and editing. **Arun D. Pherwani**: Conceptualization, supervision, writing – review and editing. **Ian Chetter**: Conceptualization, supervision, writing – review and editing. **David A. Cromwell**: Conceptualization, supervision, writing – review and editing.

## CONFLICT OF INTEREST STATEMENT

The authors declare that they do not have any competing interests.

## ETHICS STATEMENT

NHS Health Research Authority and the South Yorkshire Research Ethics Committee (22/YH/0290). All participants provided written consent.

## CONSENT FOR PUBLICATION

All data included in the manuscript is anonymised. Participants provided written consent for the use of their anonymised quotes in publications.

## Supporting information

Supporting Information S1

Supporting Information S2

## Data Availability

The datasets generated and/or analysed during the current study are not publicly available as participants were not consented for their data to be shared in this manner. Data are available from the corresponding author on reasonable request.
