# Supplementary material for: Patient experience of the process to diagnosis of chronic limb‐threatening ischaemia: A qualitative study
Source: J Foot Ankle Res. 2024 Jul 17;17(3):e12042. doi: 10.1002/jfa2.12042 (PMC11633341; doi:10.1002/jfa2.12042)
Supplement: Supplementary file 1 — Supporting Information S1 [file JFA2-17-e12042-s001.docx]

| **Domain 1: research team and reflexivity** | | |
| --- | --- | --- |
| **Personal characteristics** | | |
| 1. Interviewer/facilitator | Which author(s) conducted the interview or focus group? | One researcher (EA) conducted the interviews |
| 2. Credentials | What were the researcher’s credentials? (e.g. PhD, MD) | EA is working towards a postgraduate qualification. Her current qualifications are: BMedSci, BMBS, PGCert (Vascular Surgery), FRCS (Vasc). |
| 3. Occupation | What was their occupation at the time of the study? | EA is a vascular surgeon |
| 4. Gender | Was the researcher male or female? | EA is female |
| 5. Experience and training | What experience or training did the researcher have? | EA has experience of qualitative interviewing and analysis on different projects |
| **Relationship with participants** | | |
| 6. Relationship established | Was a relationship established prior to study commencement? | No participants were known to EA prior to the study commencement |
| 7. Participant knowledge of the interviewer | What did the participants know about the researcher? (e.g. personal goals, reasons for doing the research) | Participants knew EA’s background as a vascular surgeon, and understood the purpose of the research |
| 8. Interviewer characteristics | What characteristics were reported about the interviewer/facilitator? (e.g. bias, assumptions, reasons and interests in the research topic) | EA, the researcher who carried out interviews and analysis, is a vascular surgeon and used her subjectivity to develop themes relevant to the vascular clinician. This unique analysis is a hallmark of reflexive thematic analysis and should be embraced. |
| **Domain 2: study design** | | |
| **Theoretical framework** | | |
| 9. Methodological orientation and theory | What methodological orientation was stated to underpin the study? (e.g. grounded theory, discourse analysis, ethnography, phenomenology, content analysis) | Reflexive thematic analysis was used, carried out within a critical realist framework. |
| **Participant selection** | | |
| 10. Sampling | How were participants selected? (e.g. purposive, convenience, consecutive, snowball) | The purposive sampling strategy aimed for maximum variation in age, gender, diabetes status and patient location in the vascular network (near a hub or spoke hospital) |
| 11. Method of approach | How were participants approached? (e.g. face to face, telephone, mail, e-mail) | Participants were approached via telephone, email or face to face |
| 12. Sample size | How many participants were in the study? | 16 |
| 13. Non-participation | How many people refused to participate or dropped out? Reasons? | None dropped out or rescinded consent at a later stage |
| **Setting** | | |
| 14. Setting of data collection | Where was the data collected? (e.g. home, clinic, workplace) | Interviews were carried out over the telephone |
| 15. Presence of non-participants | Was anyone else present besides the participants and researchers? | One participant had their partner with them during the interview |
| 16. Description of sample | What are the important characteristics of the sample? (e.g. demographic data, date) | See Table 1 |
| **Data collection** | | |
| 17. Interview guide | Were questions, prompts, guides provided by the authors? Was it pilot tested? | A topic guide consisting of open questions was used as a framework for the interviews, with prompts used where necessary. It was iteratively altered as interviews progressed |
| 18. Repeat interviews | Were repeat interviews carried out? If yes, how many? | No |
| 19. Audio/visual recording | Did the research use audio or visual recording to collect the data? | Each interview was audio recorded |
| 20. Field notes | Were field notes made during and/or after the interview or focus group? | A reflexive diary was kept throughout the process by EA, with individual reflections recorded after each interview |
| 21. Duration | What was the duration of the interviews or focus group? | Interviews lasted from 38 to 61 minutes (mean 47 minutes) |
| 22. Data saturation | Was data saturation discussed? | Reflection on the content of our dataset found the interview data adequately rich to fulfil our research aim, according to the concept of information power |
| 23. Transcripts returned | Were transcripts returned to participants for comment and/or correction? | No |
| **Domain 3: analysis and findings** | | |
| **Data analysis** | | |
| 24. Number of data coders | How many data coders coded the data? | Inductive coding was carried out by EA |
| 25. Description of the coding tree | Did authors provide a description of the coding tree? | The coding was inductive and according to reflexive thematic analysis, which does not use a coding tree |
| 26. Derivation of themes | Were themes identified in advance or derived from the data? | Candidate themes were generated initially, then developed and revised following discussion with the wider author team and re-engagement with the original data. Themes were then refined, defined and named |
| 27. Software | What software, if applicable, was used to manage the data? | NVivo software, Microsoft Excel |
| 28. Participant checking | Did participants provide feedback on the findings? | No |
| **Reporting** | | |
| 29. Quotations presented | Were participant quotations presented to illustrate the themes/findings? Was each quotation identified? (e.g. participant number) | See Results - yes |
| 30. Data and findings consistent | Was there consistency between the data presented and the findings? | When reviewed against the initial transcripts, the study findings accurately reflect the data collected |
| 31. Clarity of major themes | Were major themes clearly presented in the findings? | See Results - yes |
| 32. Clarity of minor themes | Is there a description of diverse cases or discussion of minor themes? | See Results - yes |
