# Supplementary material for: Patient experience of the process to diagnosis of chronic limb‐threatening ischaemia: A qualitative study
Source: J Foot Ankle Res. 2024 Jul 17;17(3):e12042. doi: 10.1002/jfa2.12042 (PMC11633341; doi:10.1002/jfa2.12042)
Supplement: Supplementary file 2 — Supporting Information S2 [file JFA2-17-e12042-s002.docx]

**Interview topic guide: patients**

Questions are likely to include the following:

- Introduction, aims of interview, safety information, reminder that the interview is being recorded, the participants can stop at any time, reminders of any possible escalation in the event of risk disclosure
- Confirm consent
- Patient characteristics:
  - Age, gender
  - Comorbidity (diabetes)
  - Local hospital (hub or spoke)
- I understand you’ve had a diagnosis of chronic limb-threatening ischaemia in the last year. Can you tell me a bit more of your story?

**Allow participant to tell their story uninterrupted in their own words. Then go through, slowly, picking out relevant points to discuss further. Allow the participant to lead the conversation.**

- When did you first notice something wasn’t right? What symptoms were you having?
  - What did you do? Did you tell anyone?
  - What support did you need?
  - Did your symptoms affect your relationships with friends or family?
  - Did your symptoms affect your working life?
  - How did that all make you feel?
- Did you see a primary care doctor (GP), nurse or podiatrist? Did you go to A&E?
  - What did you tell them?
  - What did they tell you?
  - How did you feel?
  - Was there any delay?
- When did you first see the vascular surgery specialists at the hospital – this might have been a nurse, a podiatrist or a doctor?
  - What was that experience like?
  - Was there a delay?
  - How did you feel?
  - Did having diabetes make a difference to who you saw?
- Were you a smoker?
  - Did you feel judged for smoking?
- If spoke patient, did you travel to the hub?
- Was this an issue?
- What do you think of the hub compared to the spoke?
- When did you first see a vascular surgeon?
  - What did they tell you?
  - How did you feel?
- At what point did you understand what your diagnosis was? – A lack of blood supply putting your foot / your leg at risk.
  - - Have you heard the term “chronic limb-threatening ischaemia” or CLTI before?
    - Did you Google your symptoms?
- If your next door neighbour had the same thing, what would you tell them?
- What was good about your experience from first symptom to assessment?
- What was bad about your experience from first symptom to assessment?
  - Was there any effect of delays on your trust in medical services?
- What would matter to you the most if you were to have the same problem on the other leg?
  - Would you change anything you did the first time?
  - PROMPTS: Would keeping your independence matter to you? Would being pain free matter to you? Would keeping your leg matter to you?
- Any questions for me?
- What would you like to hear from us in the future?
  - PROMPTS: Would you like to hear the results of all the interviews? How you have helped us?
- We would love to have more people like you involved in making our services better. Would you consider being involved in future projects?

Questioning here will be adapted in response to ongoing iterative analysis of the interview data and feedback from participants.
